# Development of Inductive Current Transformer 400/5/1 of Class 0.2S for Frequency Range 50 Hz–5 kHz

**DOI:** 10.3390/s24248011

**Published:** 2024-12-15

**Authors:** Michal Kaczmarek, Blazej Pacholczyk

**Affiliations:** Institute of Mechatronics and Information Systems, Lodz University of Technology, 90-537 Lodz, Poland

**Keywords:** inductive current transformer, class 0.2S, higher harmonics, wideband transformation accuracy, current error, phase displacement, wide frequency

## Abstract

This paper is devoted to the development of a window-type inductive current transformer (iCT) with a rated primary current equal to 400 A and two secondary windings with rated currents of 5 A and 1 A. Its novelty concerns the presentation of this process in the case of an iCT with a 0.2S accuracy class ensured not only for a sinusoidal current of a frequency of 50 Hz but also for the transformation of distorted current in the harmonic frequency range from 50 Hz to 5 kHz. The maximum permissible values of the current error and phase displacement are equal to ±0.2% and ±0.2°, respectively, and are the same as the limiting values for a 50 Hz sinusoidal current of a rated RMS value. In the design process, three materials were considered for the construction of the developed iCT 400/5/1: electrical steel, permalloy and nanocrystalline (Nano). Their wideband properties were analyzed and the choice of the Nano magnetic core was justified with the already-available data in the literature on the basis of elaborated design assumptions. In order to specify its particular type, four magnetic cores with different initial magnetic permeabilities were tested. It was demonstrated that, as far as more favorable magnetic properties also involved increased active power losses in the magnetic core, it was not an appropriate choice for the construction of the iCT, especially for the transformation of the current with a main frequency equal to 50 Hz or 60 Hz.

## 1. Introduction

An inductive current transformer (iCT) is designed to operate in the near-short-circuit condition of its secondary winding. It is used to transform the high-value primary current into the relatively low-value secondary current (typically, its rated values are equal to 1 A or 5 A) required for connected measuring or protection equipment. Inductive current transformers are an essential part of electrical power infrastructure. They play an important role in the monitoring, control and protection systems of electrical networks in order to ensure the reliable operation of electrical power systems. ICTs should be designed for real operation conditions, not only for the transformation of sinusoidal current but also distorted current. Therefore, this is the aim of this paper. Their wideband transformation accuracy is required for the measurement of distorted electrical power and assessment of the power quality. This is due to the fact that electricity meters measure both distorted active and reactive power, and in order to ensure accurate measurements, the instrument transformer should ensure a high level of wideband metrological performance [1,2,3]. Measuring systems have been developed to test the wideband transformation accuracy of iCTs for distorted currents [4,5,6,7,8,9,10,11]. In the approach presented in the papers [4,5,6,10], tests were carried out under rated ampere-turn conditions. In this scenario, the step-up current transformer is not needed to provide the tested iCT with the high RMS value of the primary current to replicate the existing operating environment. However, this method is only applicable to window-type iCTs. This is because an additional primary winding has to be made with the required number of turns being the same as in the secondary winding. Such a system may be supplied directly from the wideband power amplifier. The testing of a bar-type CT requires a step-up current transformer. In this measurement setup, a reference signal source is also required (for rated ampere-turns, this would be the primary current in the additional primary winding). To provide this, specially designed wideband inductive CTs or electronic transducers may be utilized [12,13,14,15].

The transformation accuracy of distorted currents by iCTs is most significantly affected by the phenomenon of the self-generation of lower-order higher harmonics. It causes an increase in the values of the current error and phase displacement of their transformation as a result of the additional self-distortion of the secondary current, due to the non-linearity of the magnetization characteristics of the magnetic core [5,6]. This is the main reason for the problems of maintaining the same accuracy class for higher harmonics as ensured for the transformation of the 50 Hz sinusoidal current by typical manufactured iCTs. This phenomenon means that, even for such operation conditions, lower-order higher harmonics are present in the secondary current of iCTs, while for distorted current, it affects the RMS values and phase shifts in corresponding-order higher harmonics. Moreover, an increase in the maximum value of the magnetic flux density in the magnetic core causes an increase in the self-generation of the low-order higher harmonics and the deterioration of iCTs’ transformation accuracy. This is associated with an increase in the load impedance connected to the secondary winding and/or an increase in the RMS value of the primary current. To enhance the wide-frequency transformation characteristics of iCTs, compensation techniques may be employed [16,17,18,19,20,21]. This may involve the utilization of active components to control the secondary current or voltage and to generate low-order higher harmonics that are in counterphase with the self-generated low-order higher harmonics caused by the nonlinearity of the magnetization characteristic of the magnetic core. Therefore, their RMS values and phase angles are required to be determined from measurements performed for the transformation of sinusoidal and distorted current under varying loads of the secondary winding and different RMS values of the primary-current harmonics. However, this approach has a significant drawback; the resulting matrix of measured data is large and requires a high number of test points. Moreover, this issue becomes more pronounced if high transformation accuracy is required and the influence of the low-order higher harmonics from the distorted primary current needs to be considered [5,6].

The aim of this article is to present the development process of a wideband, window-type iCT for a rated RMS primary current equal to 400 A. Its application requires two secondary windings for rated currents of 5 A and 1 A; an accuracy class of 0.2S must be ensured in the entire frequency range from 50 Hz to 5 kHz for resistive loads from 1 W to 5 W. It should be noted that in the standard IEC 61869-1, optional wideband accuracy classes for iCTs are defined [22]. However, the most restrictive requirements of 0.2S-WB2 for this frequency range are ±2%/±2° [22,23]. The developed iCT was designed for transformation accuracy with the maximum permissible values of the current error and phase displacement being ±0.2% and ±0.2°, the same as is required for the 50 Hz sinusoidal current of rated RMS values by the standard IEC 61869-2 [24]. Its design involved the consideration of the influence of the magnetic flux density in the magnetic core as the main factor that conditions its transformation accuracy. The results indicate that the secondary winding load conditions and the maximum value of its distorted current determines the secondary voltage and the maximum value of the magnetic flux density in the magnetic core. Therefore, the values of the current error and phase displacement of the iCT, for the magnetic core of a given relative permeability, results from a fixed operating point in its magnetization characteristic by the maximum value of the secondary voltage. This is due to the fact that required value of the magnetic core excitation current and the magnetizing current as well as current associated with the active power losses in the magnetic core results from the maximum value of the magnetic flux density in the magnetic core. Since the transformation accuracy of iCTs for distorted current is most significantly affected by the self-distortion of the secondary current to minimize the RMS values of the self-generated (into the secondary current of iCTs) low-order harmonics, it is required to make the cross-section of the magnetic core oversized in relation to its typically used size for the same rated load of the secondary winding. Moreover, the diameter of the secondary winding wire should also be oversized in order to decrease its resistance. Therefore, it is required that the lowest possible operating point of an iCT is ensured in its non-linear magnetization characteristic: the lowest possible magnetic flux density in the magnetic core. The contribution of this paper concerns its demonstration of the design process of an iCT optimized for wideband transformation and high accuracy for sinusoidal as well as distorted current.

## 2. Principle of Inductive Current Transformers

**Figure 1:**
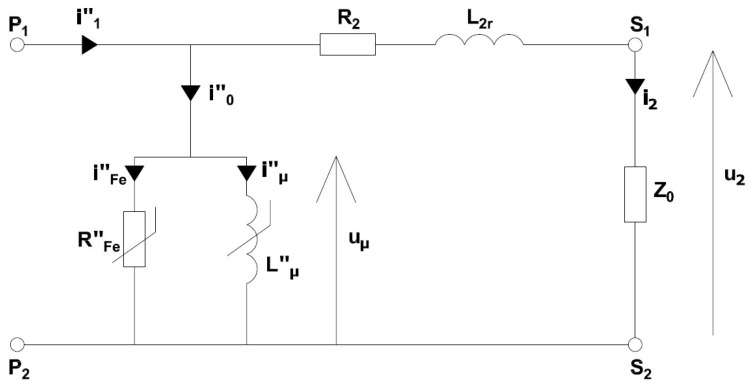
The equivalent circuit of an iCT.

An iCT consists of the following main components: a magnetic core, primary and secondary windings and an insulation system (MV and HV constructions). The active parts of a medium-voltage current transformer are usually insulated with epoxy resin or paper-oil. In a window-type device, there is no primary winding and the primary conductor is led by the window of the magnetic core. In the case of a wound-type iCT, the primary terminals are used to connect a bus or cable to the primary winding. These terminals are marked with the capital letters P1 and P2. The magnetic core of an iCT is a key element in its design. It should be made of a material with a high level of initial magnetic permeability. Its primary and secondary circuits are connected through a common magnetic flux. A magnetic core may be made from a variety of materials depending on the specific requirements of the application, e.g., electrical steel, permalloy, nanocrystalline or amorphous materials. The secondary winding usually consists of a copper wire wound on a magnetic core. Induced voltage between its terminals results from the magnetic flux in the magnetic core being the difference between the magnetic flux from the primary winding and the secondary winding, as well as from its number of turns. The secondary terminals are marked with the capital letters S1 and S2. The earth terminal is used to force the earth potential onto the transformer housing. In Figure 1, the equivalent circuit of an iCT is presented.

The following notations are used in Figure 1 (symbols with ″ represent quantities converted into the secondary side of the iCT):

$P_{1/2}$—primary winding terminals;
$S_{1/2}$—secondary winding terminals;
$i_{0}^{\prime \prime }$—the instantaneous value of the magnetic core excitation current;
$i_{1}^{\prime \prime }$—the instantaneous value of the primary current;
$i_{2}$—the instantaneous value of the secondary current;
$i_{Fe}^{\prime \prime }$—the instantaneous value of the current associated with the active power losses in the magnetic core;
$i_{\mu }^{\prime \prime }$—the instantaneous value of the magnetic core magnetizing current;
$u_{2}$—the instantaneous value of the secondary voltage;
$R_{Fe}^{\prime \prime }$—the equivalent resistance of the magnetic core;
$L_{\mu }^{\prime \prime }$—the mutual inductance of the iCT’s windings;
$L_{2r}$—the leakage inductance of the secondary winding;
$R_{2}$—the resistance of the secondary winding;
$u_{\mu }$—the instantaneous value of the voltage with an equivalent mutual inductance in the transformer winding;
$Z_{0}$—the impedance of the secondary winding load composed of R_0_ (resistance) and L_0_ (inductance).

In the equivalent circuit presented in Figure 1, the primary winding resistance and leakage inductance are omitted as they do not have any effect on the operation of the iCT. Its secondary current may be calculated from the following equation:

(1) $$i_{2}=i_{1}^{\prime \prime }-i_{0}^{\prime \prime }$$

Therefore, the non-ideal magnetic core is the source of transformation errors in the iCT.

The magnetic flux passing through a given surface can be determined according to the following relationship:

(2) $$\Phi_{0}=\iint_{S}\vec{B}\hat{n}dS$$

where

S—surface area;
$\vec{B}$—magnetic flux density;
$\hat{n}$—the unit vector normal to the surface.

The magnetic flux $\Phi_{0}$ is multiplied by the number of winding turns.

(3) $$\Phi_{0}=z\cdot \Psi$$

where

*Ψ*—the magnetic flux associated with a single turn of the winding;
*z*—the number of winding turns.

In accordance with Faraday’s law, the electromotive force (EMF) may be calculated from Equations (4) and (5):

(4) $$e_{1}=-z_{1}\frac{d\Psi }{dt}=-L_{µ}\frac{{di}_{µ}}{dt}$$

(5) $$e_{2}=-z_{2}\frac{d\Psi }{dt}$$

(6) $$e_{1}^{\prime }=e_{2}=-u_{\mu }$$

where

$e_{1}$—the induced electromotive force (EMF) in the primary winding;
$e_{2}$—the induced electromotive force (EMF) in the secondary winding;
$z_{1}$—the number of turns in the primary winding;
$z_{2}$—the number of turns in the secondary winding.

The maximum value of the magnetic flux density in the magnetic core of the iCT may be expressed by the following equation [6]:

(7) $$B_{m}=max\{\frac{1}{z_{2}\cdot s_{Fe}}\int u_{2}\left(t\right)dt\}$$

In the case of a sinusoidal current of the frequency *f*, for the equivalent circuit presented in Figure 1, Formula (7) may be simplified to the following:

(8) $$B_{m}\approx max\{\frac{i_{2}\cdot \sqrt{{\left(R_{2}+R_{0}\right)}^{2}+{\left({2\cdot \pi \cdot f\cdot L}_{2r}+{2\cdot \pi \cdot f\cdot L}_{0}\right)}^{2}}}{4.44\cdot f\cdot z_{2}\cdot S_{Fe}}\}$$

Ampère’s law expresses the relationship between the magnetizing current and the intensity of the magnetic field strength in the magnetic core:

(9) $$H_{max}=\frac{i_{\mu }^{"}\cdot z_{1}}{l_{Fe}}$$

where

$H_{max}$—the magnetic field strength in the magnetic core;
$l_{Fe}$—the average length of the magnetic flux path in the magnetic core.

The relative magnetic permeability of the magnetic core may be expressed by the following equation:

(10) $$\mu =\frac{B_{max}}{H_{max}}$$

where

μ—the relative magnetic permeability of the magnetic core.

**Figure 2:**
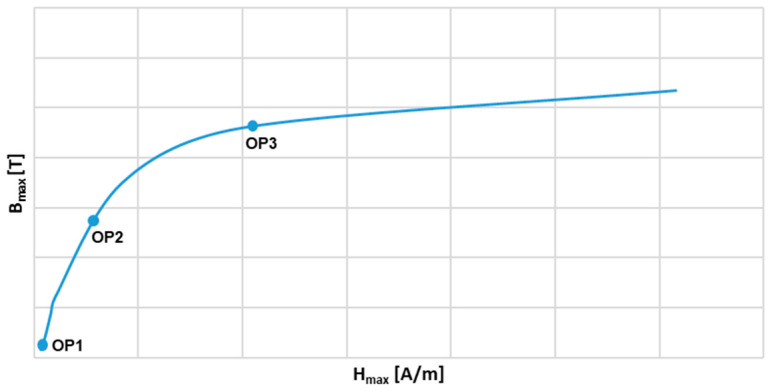
An example of the magnetization characteristic of an iCT’s magnetic core with three operating points in its distinctive regions.

The dependencies in (8) indicate that the secondary winding load value and its power factor as well as the value of the secondary current (the secondary voltage *u*_2_) determine the maximum value of the magnetic flux density in the magnetic core. Therefore, the values of the current error and phase displacement of the iCT, for the magnetic core of a given relative permeability μ, results from this operating point in its magnetization characteristic and the required values of the magnetizing current $i_{\mu }^{\prime \prime }$ as well as the current $i_{Fe}^{\prime \prime }$ (associated with the active power losses in the magnetic core). In Figure 2, an example of the magnetization characteristic of an iCT’s magnetic core with three operating points in its distinctive regions is presented.

In Figure 2, three operating points in the magnetization characteristic of the iCT magnetic core are selected:

OP1: in the initial region;
OP2: in the quasi-linear region;
OP3: in the saturation region.

**Figure 3:**
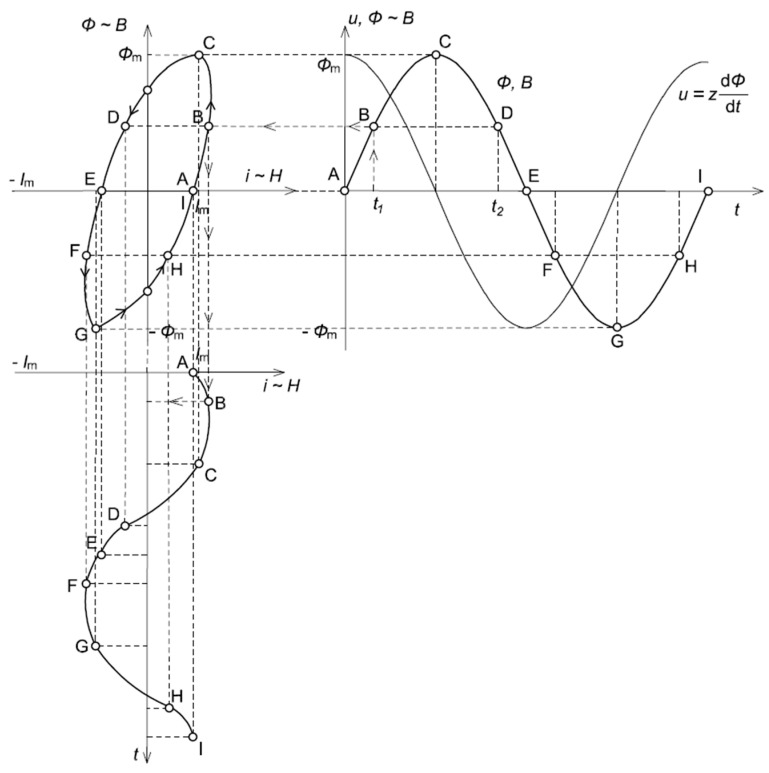
Analysis of the influence of a hysteresis loop on the distortion of the magnetizing current in the iCT’s operating point in the quasi-linear region of the magnetization characteristic.

In Figure 3, an analysis of the influence of a hysteresis loop on the distortion of the magnetizing current for the iCT’s operating point in the quasi-linear region of the magnetization characteristic is presented.

It can be seen from Figure 3 that when the magnetic core operates in the quasi-linear region of the magnetization characteristic, the magnetizing current is approximately sinusoidal. The self-generation problem of the low-order higher harmonics into the secondary current of the iCT is negligible.

**Figure 4:**
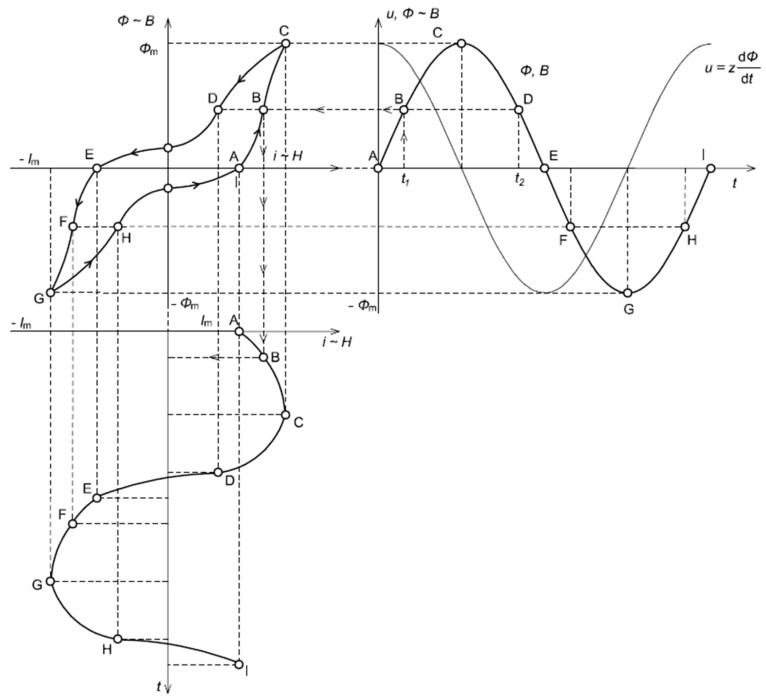
Analysis of the influence of the hysteresis loop on the distortion of the magnetizing current for the iCT’s operating point in the initial region of the magnetization characteristic.

In Figure 4, the analysis of the influence of the hysteresis loop on the distortion of the magnetizing current for the iCT’s operating point in the initial region of the magnetization characteristic is presented.

Due to the non-linearity of the magnetization characteristic of the iCT’s magnetic core and deformation of the hysteresis loop from an elliptical shape, the problem of the low-order higher harmonics’ self-generation into the secondary current may become noticeable when the iCT operating point is in the initial region of the magnetization characteristic.

**Figure 5:**
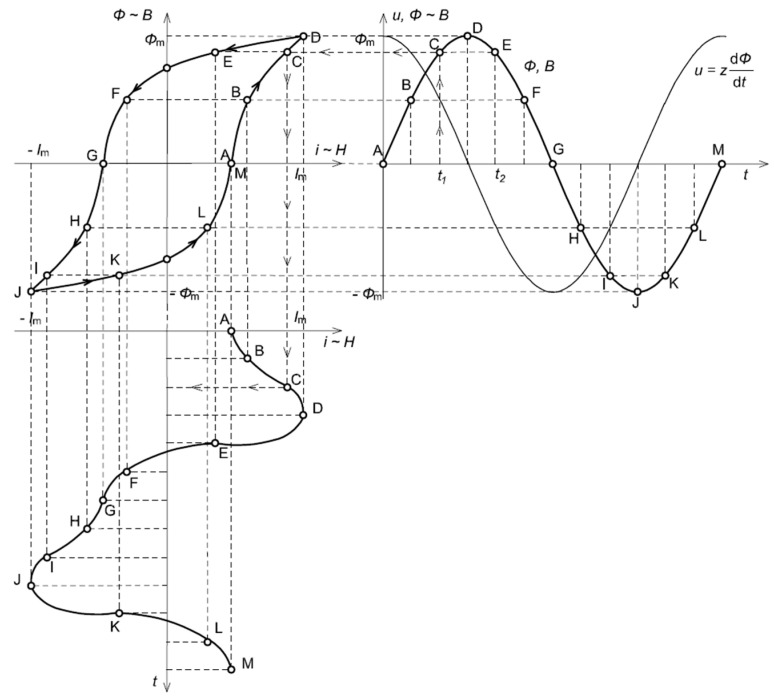
Analysis of the influence of the hysteresis loop on the distortion of the magnetizing current in the iCT’s operating point in the saturation region of the magnetization characteristic.

In Figure 5, the analysis of the influence of the hysteresis loop on the distortion of the magnetizing current for the iCT’s operating point in the initial region of the magnetization characteristic is presented.

It can be seen from Figure 5 that when the magnetic core operates in the saturation region of the magnetization characteristic, the magnetizing current is non-sinusoidal. The problem of the low-order higher harmonics’ self-generation into the secondary current of the iCT is significant. Their RMS values increase, while the operating point of the iCT, due to the increase in the secondary winding load and/or RMS value of the primary current, moves from the linear region to the saturation region. The increase in the RMS values of the harmonics is reduced with the increase in their frequency.

## 3. Requirements for Wideband Operation and Selection Process of iCT’s Magnetic Core

In accordance with the standard IEC 61869-2, the current error of the iCT is defined by the following equation [24]:

(11) $$∆I=\frac{k_{I}I_{2}-I_{1}}{I_{1}}\cdot 100\%$$

where ∆I is the current error, $I_{1}$ is the RMS value of the primary current, $I_{2}$ is the RMS value of the secondary current and $k_{I}$ is the rated current ratio.

In accordance with the standard IEC 61869-2, the phase displacement of the iCT is defined by the following equation [24]:

(12) $$\delta \varphi =\varphi_{2}-\varphi_{1}$$

where δφ is the phase displacement, $\varphi_{1}$ is the phase angle of the primary current in relation to the reference and $\varphi_{2}$ is the phase angle of the secondary current in relation to the reference.

The composite error is the percentage value resulting from the effective value of the difference between the instantaneous values of the primary and secondary currents (after multiplication by the nominal transformation ratio) in relation to the effective value of the primary current. In accordance with the standard IEC 61869-2, it is defined by the following equation [24]:

(13) $$\varepsilon_{I\%}=\frac{100}{I_{1}}\sqrt{\frac{1}{T}\int_{0}^{T}{\left(k_{I}i_{2}-i_{1}\right)}^{2}dt}=\frac{I_{0}}{I_{1}}\cdot 100\%$$

where $\varepsilon_{I\%}$ is the composite error and T is the period resulting from the frequency of the main component of the primary current.

Therefore, it may be also determined from the values of the current or voltage error and phase displacement [6,9]:

(14) $$\varepsilon_{I\%}=\sqrt{{∆I}^{2}+{sin(\delta \varphi \cdot 100\%)}^{2}}$$

where $\varepsilon_{I\%}$ is the composite error and T is the period resulting from the frequency of the main component of the primary current.

In Table 1, the limiting values of the current error and phase displacement in accordance with the standard IEC 61869-2 for each defined accuracy class of the iCT are presented [24].

The presented accuracy classes of the iCT are determined for the transformation of a sinusoidal current of a frequency of 50 Hz or 60 Hz. The requirement for the designed iCT is to maintain the same accuracy class and not to exceed the limiting values defined for class 0.2S for the transformation of all harmonics of the distorted primary current from a main frequency of 50 Hz up to 5 kHz. It should be noted that in the standard IEC 61869-1, optional wideband accuracy classes for iCTs are defined. However, the requirements for 0.2S-WB2 for this frequency range are ±2%/±2° [22]. Therefore, a more restrictive and simplified approach was adopted.

**Figure 6:**
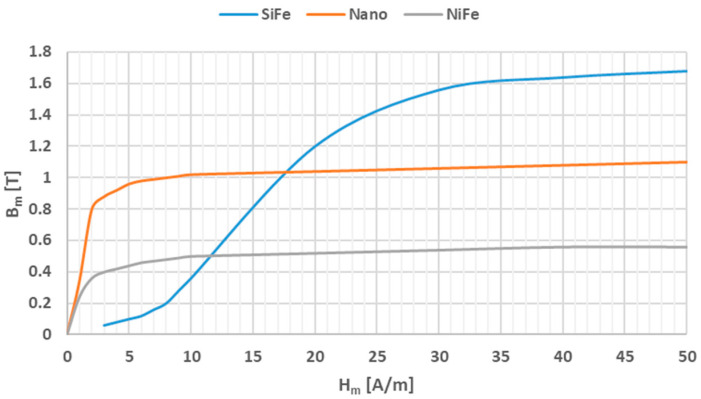
Example of magnetization characteristics of toroidal cores made of SiFe, Nano and NiFe based on data presented in [29].

Three materials were considered for use for the construction of the developed iCT 400/5/1, class 0.2S, for the frequency range 50 Hz–5 kHz: electrical steel (SiFe), permalloy (NiFe) and nanocrystalline (Nano) [6,25,26,27,28]. Electrical steel consists mainly of iron with a small addition of silicon (about 5%), while permalloy consists of about 80% nickel and about 20% iron. The nanocrystalline material (e.g., Fe-Si-B-Nb-Cu) is composed of about 80% iron and silicon, boron, niobium and copper. The silicon is added to reduce the active power losses associated with magnetization. SiFe is characterized by a high saturation magnetic flux density. It is therefore used in the manufacture of iCTs for protection and measurement when high transformation accuracy is not required. Permalloy has a significantly lower saturation magnetic flux density and active power losses as well as higher magnetic permeability. Consequently, this material is used for the production of iCTs for measuring when high transformation accuracy and a low safety factor (SF) are required. Figure 6 shows an example of the magnetization characteristics of electrical steel (SiFe), permalloy (NiFe) and nanocrystalline (Nano) magnetic cores (based on data presented in the article [29]).

Figure 6 indicates that for the SiFe core, a magnetic field strength of approximately 8 A/m is required to achieve a maximum magnetic flux density of 0.2 T, while in the case of the NiFe or the Nano core, only about 0.8 A/m is required. Therefore, the iCT will have smaller transformation errors because of a lower magnetizing current at the same operation point resulting from the load of the secondary winding at a given primary current. Moreover, the Nano magnetic core is characterized by an initial saturation magnetic flux density equal to about 0.8 T, whereas for the NiFe core, it is only 0.25 T. This indicates about a three-times-higher potential load possibility in the secondary winding in the quasi-linear region of the magnetization characteristic, with the best transformation accuracy ensured.

**Figure 7:**
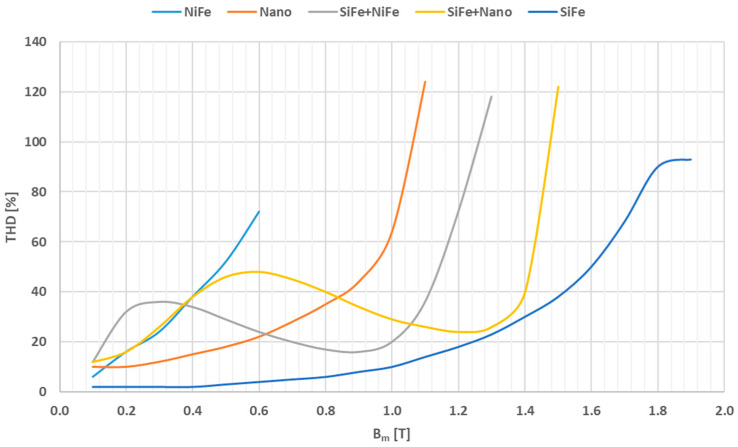
The THD of the magnetizing current regarding the maximum flux density in the magnetic core based on data presented in [29].

In Figure 7, the dependence of the total harmonic distortion (THD) of the magnetizing current regarding the maximum magnetic flux density in the magnetic core is presented based on data provided in the article [29].

The SiFe magnetic core is characterized by the lowest THD of the magnetizing current. However, this example shows how important is the interpretation of measurement results in terms of the possible application of a given material for iCT construction. In this case, the low value of the THD factor indicates that SiFe is characterized by the high value of the fundamental component of the magnetizing current, and for this reason, it does not possess favorable properties for accurate distorted-current transformation.

**Figure 8:**
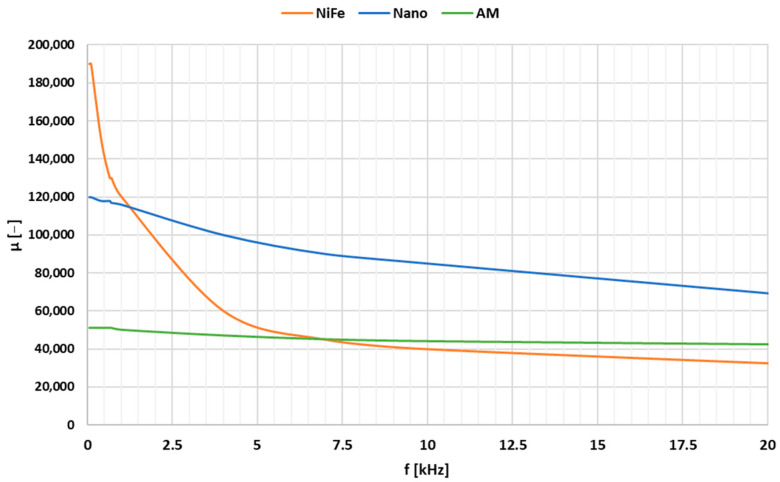
The change in the initial relative magnetic permeability regarding the frequency of the magnetizing current of the NiFe, Nano and amorphous (AM) magnetic cores based on data from [30].

Figure 8 shows an example of the change in the initial relative magnetic permeability with the frequency of the magnetizing current of the NiFe, Nano and amorphous (AM) magnetic cores, based on data presented in the article [30].

The results from Figure 8 indicate that the increase in the frequency of the magnetizing current, e.g., from 50 Hz to 5 kHz, causes a decrease in the initial relative magnetic permeability of the NiFe magnetic core from about 190,000 to 50,000 (depending on its type), while in the case of the Nano material, the decrease is from about 120,000 to 90,000. The amorphous (AM) magnetic core shows a lower change in permeability with frequency but is characterized by a lower initial relative magnetic permeability value for a low-frequency magnetizing current, e.g., 50 Hz. Therefore, for the construction of the wideband iCT, a magnetic core made of the nanocrystalline material was chosen. In order to specify its type, four models of the iCT were made of Nano materials with the initial magnetic permeabilities of 40,000, 100,000, 150,000 and 200,000. In Table 2, their characterization is presented.

**Figure 9:**
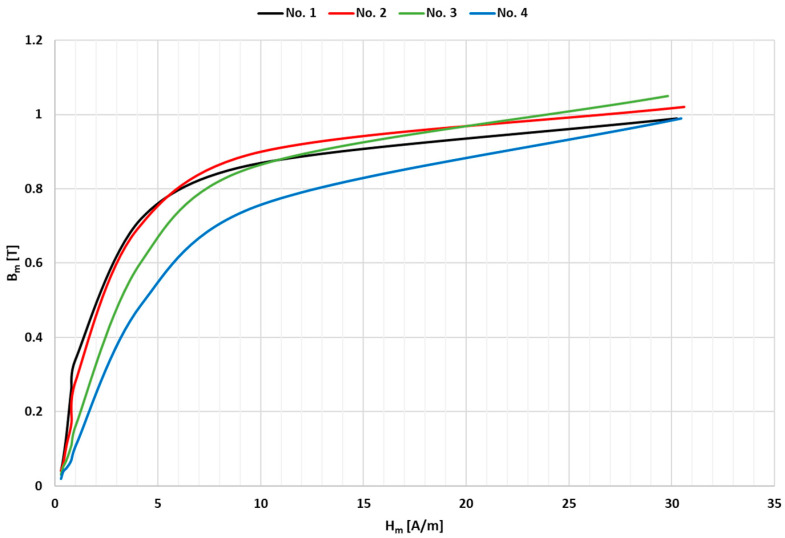
Magnetization characteristics of toroidal nanocrystalline magnetic cores with initial permeabilities of 200,000, 150,000, 100,000 and 40,000 used for construction of 4 tested iCT models.

Figure 9 shows the magnetization characteristics of the toroidal nanocrystalline magnetic cores used for the construction of four tested window-type iCT models.

The comparison of the magnetization characteristics presented in Figure 9 shows that the magnetic properties of core no. 4, which has an initial permeability of 40,000, are least favorable. This is due to the smaller slope of the magnetization curve, which indicates a lower magnetic permeability over the entire operating range and a higher value of potential transformation errors of in the designed wideband iCT.

**Figure 10:**
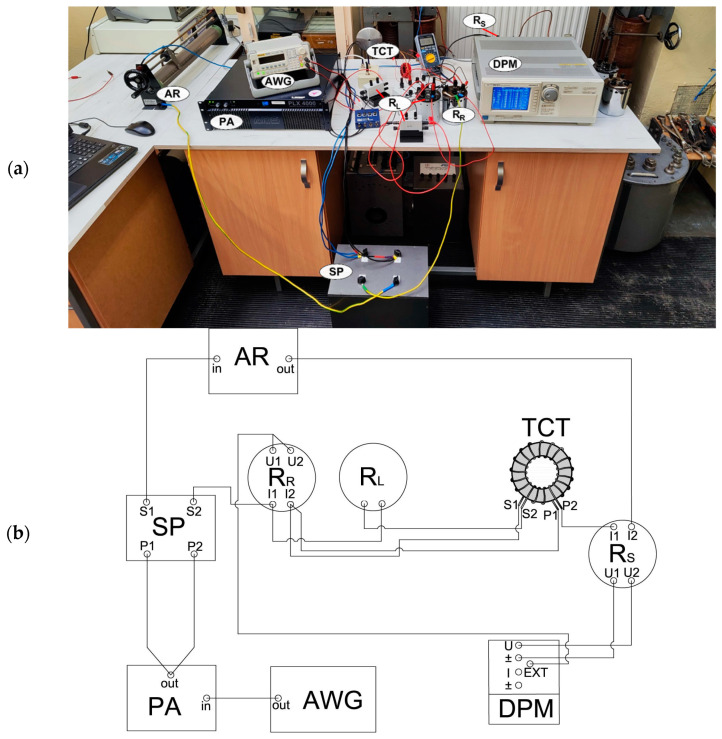
Measurement setup for testing distorted-current transformation accuracy of iCTs in rated ampere-turns conditions: (**a**) photo and (**b**) simplified electrical diagram.

The transformation accuracy of the iCT models for the harmonics of distorted current was tested in rated ampere-turns conditions. Therefore, an additional winding with 50 turns was made with a rated RMS value of a current equal to 5 A. These test conditions were equivalent to the normal operation state, with the rated primary current equal to 250 A in the single primary wire passing through the window of the magnetic core. The measurement setup is presented in Figure 10.

In Figure 10, the following notation is used:

SP—the separating transformer;
AWG—the arbitrary waveform generator;
PA—the power amplifier;
AR—the additional load resistor required for PA;
TCT—the tested iCT;
DPM–the digital power meter (both WT 1600 and WT 5000 were used);
R_R_—a 1 Ω current shunt;
R_S_—a 0.1 Ω (for 5 A and 6 A)/1 Ω (for 0.25 A)/10 Ω (for 0.5 A) current shunt selected depending on the RMS value of the measured current in the additional primary winding;
R_L_—a 0.2 Ω (5 VA)/0.1 Ω (2.5 VA) load resistor in the iCT’s secondary winding.

Tests were carried out with the power factor of the secondary winding load equal to 1 (the inductance of the load for the higher harmonics of the transformed current caused an increase in the iCT’s operating point in the magnetization characteristic towards saturation and a decrease in its transformation accuracy) [31]. One channel of the arbitrary waveform generator was used for the generation of a sinusoidal voltage of a frequency of 50 Hz, while the second channel supplied the test system with a sinusoidal voltage of a selected frequency between 100 Hz and 5 kHz. Both voltages were applied simultaneously to the audio power amplifier to feed the additional winding of the tested iCT with the following required currents: 5%, 20%, 100% and 120% of its rated RMS value, equal to 250 A (5 A with 50 turns in the additional primary winding as the ampere-turns method was used). The evaluation of the iCTs’ transformation accuracy was performed for a distorted current with a fundamental component of a frequency of 50 Hz and a single higher harmonic of an order of 3, 5, 7, 10, 20, 50, 80 or 100. Their RMS values were set to 10% of the first harmonic. The digital power meter/power analyzer was used to measure:

- The RMS values of the fundamental and higher harmonics of the current in the additional primary winding of the tested window-type iCTs from the voltage of the current shunt R_S_;
- The RMS values of the fundamental and higher harmonics of the current in the differential connection between the additional primary winding and secondary winding of the tested iCTs from the voltage of the current shunt R_R_;
- The phase angles between a given order of harmonics from both voltages from the current shunts.

The value of the k-order distorted-current harmonic in the additional primary winding of the tested iCTs was calculated from the following equation:

(15) $$I_{1ah\%}=\frac{U_{Shk}}{R_{S}}\cdot 100\%$$

where *R_S_* is the resistance of the current shunt *Rs* used to measure the current in the additional primary winding and *U_Shk_* is the RMS value of the k-order harmonic of the voltage from the current shunt *R_S_*, proportional to the current in the additional primary winding.

The value of the composite error for the transformation of the k-order distorted-current harmonic in the tested iCTs was calculated from the following equation:

(16) $$\varepsilon_{Ih\%}=\frac{U_{Rhk}\cdot R_{S}}{R_{R}\cdot U_{Shk}}\cdot 100\%$$

where *R_R_* is the resistance of the current shunt *R_R_* used to measure the differential current between the primary and secondary windings of the tested iCTs, and *U_Rhk_* is the RMS value of the k-order harmonic of the voltage from the current shunt *R_R_*_,_ proportional to the differential current.

The value of the k-order distorted-current harmonic in the secondary winding of the tested iCTs was calculated from the following equation (as the difference between the primary and secondary currents of the iCTs is equal to the value of the composite error [9]):

(17) $$I_{2h\%}=\sqrt{{I_{1ah\%}}^{2}+{\varepsilon_{Ih\%}}^{2}-2\cdot I_{1ah\%}\cdot \varepsilon_{Ih\%}\cdot \cos\theta_{Rhk}}$$

where *θ_Rhk_* is the phase angle between the k-order harmonic of voltages from the current shunts *R_R_* and *R_S_*.

The value of the current error was calculated from the following equation:

(18) $${∆I}_{hk}=\frac{I_{2h\%}-I_{1ah\%}}{I_{1ah\%}}\cdot 100\%$$

The value of the phase displacement was calculated from the following equation:

(19) $$\varphi_{hk}=arcsin\left(\frac{\sqrt{{\varepsilon_{Ih\%}}^{2}-I_{hk}^{2}}}{100\%}\right)$$

**Figure 11:**
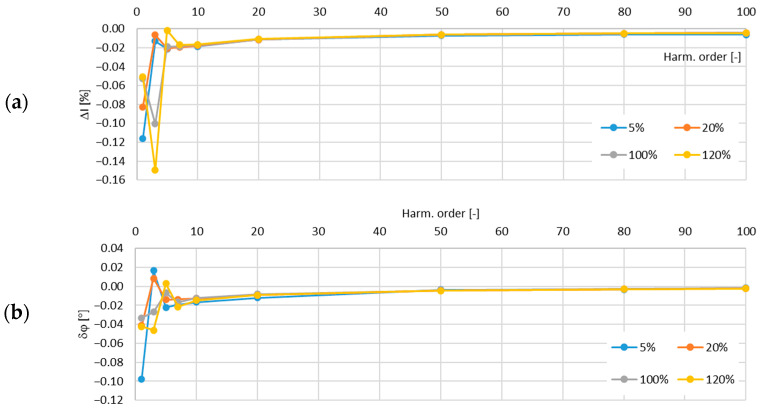
Determined values of current error (**a**) and phase displacement (**b**) of distorted-current harmonics’ transformation by the no. 1 model of iCT with secondary winding load of 5 VA.

In Figure 11, the determined values of the current error and phase displacement of the distorted-current harmonics’ transformation by the no. 1 model of the iCT with a secondary winding load of 5 VA are presented.

**Figure 12:**
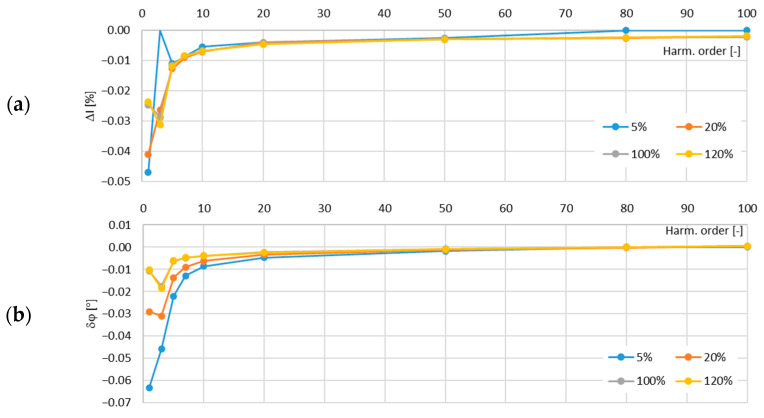
Determined values of current error (**a**) and phase displacement (**b**) of distorted-current harmonics’ transformation by the no. 1 model of iCT with secondary winding load of 1 VA.

In Figure 12, the determined values of the current error and phase displacement of the distorted-current harmonics’ transformation by the no. 1 model of the iCT with a secondary winding load of 1 VA are presented.

The analysis of the results shows that the main factor influencing the increase in the values of the transformation errors of the distorted as well as the sinusoidal current was the increase in the load of the secondary winding. This was due to the fact that for a higher magnetic flux density in the magnetic core, a higher magnetic field strength was required and so the magnetizing current increased. Moreover, the active power losses were also increased with the increase in the magnetic flux density in the magnetic core and so the excitation current was increased. The problem with the self-generation of the low-order higher harmonics into the secondary current also intensified. The increase in the frequency of the transformed harmonic caused a decrease in the transformation errors as the magnetic flux density associated with this component was reduced (in a tested frequency range up to 5 kHz). The maximum value of the current error was −0.15% for the third harmonic and that of the phase displacement was −0.1° for the main component, both detected for the secondary winding’s rated load in the tested no. 1 model of the iCTs. The results of the above analysis allow us to conclude that generated lower-order harmonics significantly increased the values of the current error (and phase displacement depending on the phase angle of the transformed harmonic in relation to the self-generated harmonic of the same order). Therefore, the operating point of the iCT in the non-linear magnetization characteristic of its magnetic core had the greatest influence on the transformation accuracy of the distorted current in the tested frequency range.

**Figure 13:**
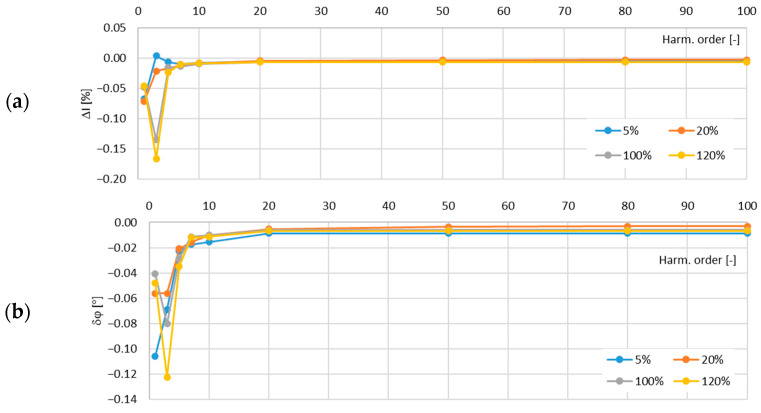
Determined values of current error (**a**) and phase displacement (**b**) of distorted-current harmonics’ transformation by the no. 2 model of iCT with secondary winding load of 5 VA.

In Figure 13, the determined values of the current error and phase displacement of the distorted-current harmonics’ transformation by the no. 2 model of the iCTs with a secondary winding load of 5 VA are presented.

**Figure 14:**
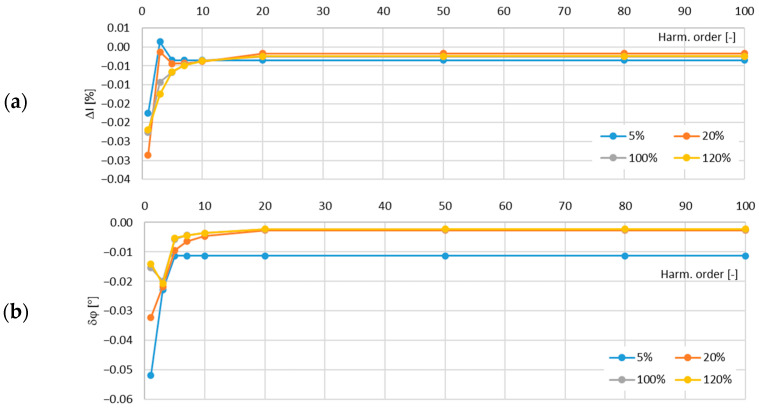
Determined values of current error (**a**) and phase displacement (**b**) of distorted-current harmonics’ transformation by the no. 2 model of iCT with secondary winding load of 1 VA.

In Figure 14, the determined values of the current error and phase displacement of the distorted-current harmonics’ transformation by the no. 2 model of the iCTs with a secondary winding load of 1 VA are presented.

The maximum value of the current error was −0.17% and that of the phase displacement was −0.13° for the third harmonic, both detected for the secondary winding rated load of the tested no. 2 model of the iCTs.

**Figure 15:**
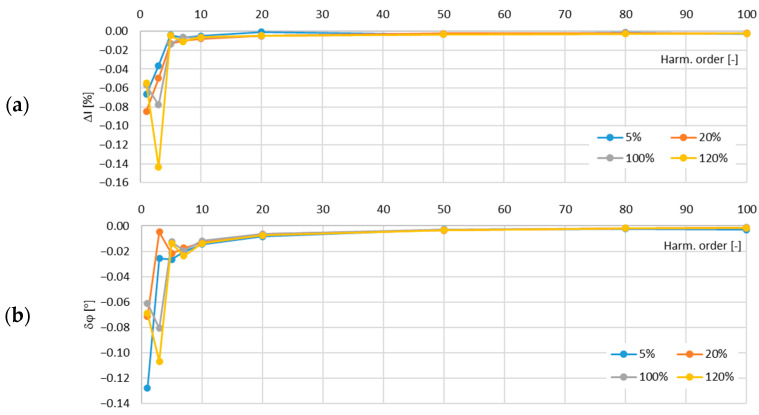
Determined values of current error (**a**) and phase displacement (**b**) of distorted-current harmonics’ transformation by the no. 3 model of iCT with secondary winding load of 5 VA.

In Figure 15, the determined values of the current error and phase displacement of the distorted-current harmonics’ transformation by the no. 3 model of the iCTs with a secondary winding load of 5 VA are presented.

**Figure 16:**
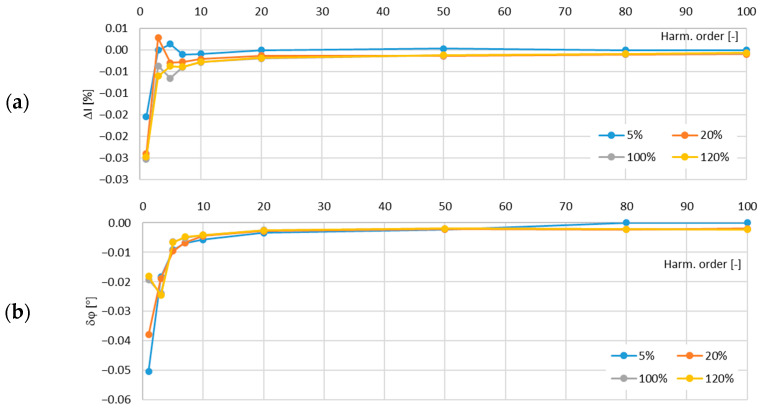
Determined values of current error (**a**) and phase displacement (**b**) of distorted-current harmonics’ transformation by the no. 3 model of iCT with secondary winding load of 1 VA.

In Figure 16, the determined values of the current error and phase displacement of the distorted-current harmonics’ transformation by the no. 3 model of the iCTs with a secondary winding load of 1 VA are presented.

The maximum value of the current error was −0.15% for the third harmonic and that of the phase displacement was −0.05° for the main component, both detected for the secondary winding rated load of the tested no. 3 model of the iCTs.

**Figure 17:**
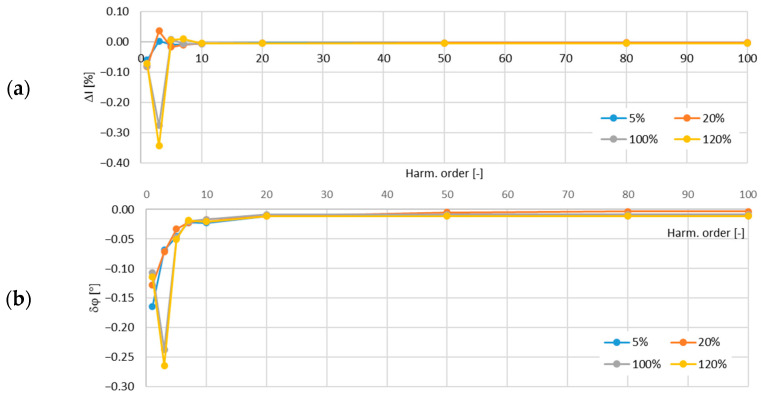
Determined values of current error (**a**) and phase displacement (**b**) of distorted-current harmonics’ transformation by the no. 4 model of iCT with secondary winding load of 5 VA.

In Figure 17, the determined values of the current error and phase displacement of the distorted-current harmonics’ transformation by the no. 4 model of the iCTs with a secondary winding load of 5 VA are presented.

**Figure 18:**
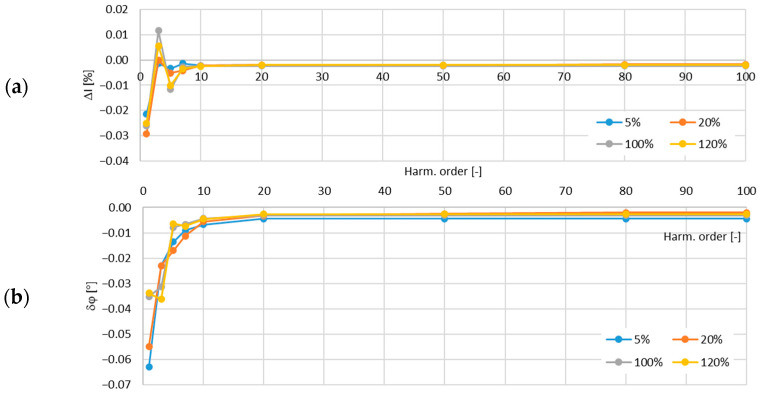
Determined values of current error (**a**) and phase displacement (**b**) of distorted-current harmonics’ transformation by the no. 4 model of iCT with secondary winding load of 1 VA.

In Figure 18, the determined values of the current error and phase displacement of the distorted-current harmonics’ transformation by the no. 4 model of the iCTs with a secondary winding load of 1 VA are presented.

The maximum value of current error was −0.35% and that of the phase displacement was −0.27° for the third harmonic, both detected for the secondary winding rated load of the tested no. 4 model of the iCTs.

The results of the studies carried out over the four iCT models allowed us to select the most appropriate magnetic core with the initial magnetic permeability of 100,000 for the construction of the designed wideband iCT 400A/5A/1A. Its advantages in terms of transformation accuracy were clearly visible for the fundamental component of distorted current. However, for higher harmonics, it showed similar properties to the magnetic cores used in the iCT models no. 1 and 2. It should be noted that the initial magnetic permeability of 200,000 involved increased active power losses in the magnetic core.

## 4. Development of Secondary Winding and Verification of Designed iCT 400A\5A\1A Transformation Accuracy for Distorted Currents

To calculate the minimal diameter of the winding wire required for the secondary winding, the following equation was used:

(20) $$D_{SW}=1.13\cdot {\left(\frac{I}{J}\right)}^{\frac{1}{2}}$$

where

$D_{SW}$—the diameter of the winding wire;
I—the rated secondary winding current;
J—permissible current density.

A current density of 6 A/mm^2^ was adopted for the calculation of the minimum diameter of the copper wire for the secondary winding. Therefore, for the rated current equal to 5 A, the required diameter of the winding wire is 1 mm, and for 1 A, it is 0.45 mm.

In the case of the designed window-type iCT, for the 400 A rated primary current, 80 turns in the secondary winding with a rated current equal to 5 A are required to be made, while 400 is necessary for the secondary winding with a rated current equal to 1 A.

**Figure 19:**
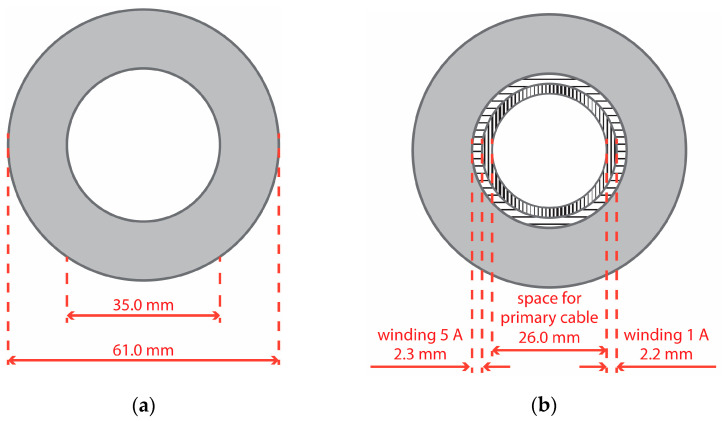
Cross-sections of the (**a**) magnetic core and (**b**) designed iCT with two secondary windings with 5 A and 1 A rated currents.

The space required for the 5 A secondary winding on the surface of the magnetic core is equal to about 90 mm, also taking into consideration the technical limitations of its manual winding (in the available 110 mm internal circumference of the magnetic core for winding turns in the single layer). To calculate the space available for the next 1 A secondary winding, a reduction in the magnetic core’s internal radius from 17.5 mm to 15.5 mm was considered. Therefore, about 100 mm of space in the already-made 5 A secondary winding is available for winding turns in the single layer. The space required for the 1 A secondary winding on the surface of the 5 A secondary winding is equal to about 200 mm, also taking into consideration the technical limitations of its manual winding. Therefore, two layers must be made. In Figure 19, the discussed dimensions of the designed iCT are presented.

According to the datasheets, the minimum cable diameter that can be used for the 400 A rated current is 22 mm. Therefore, it is possible to meet this requirement with the designed 400A/5A/1A iCT made from the magnetic core with the proposed dimensions.

**Figure 20:**
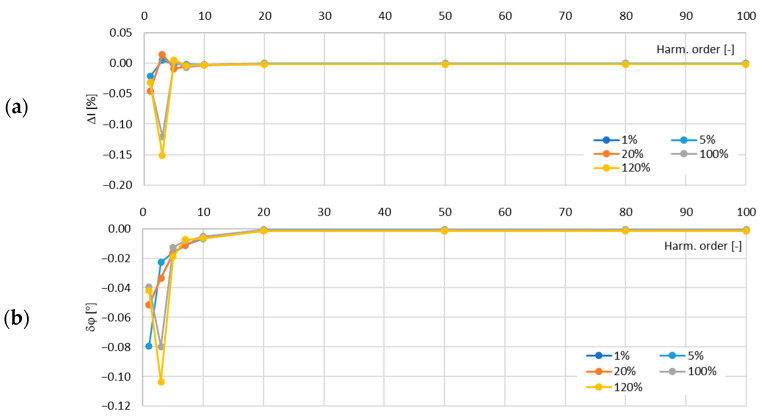
Determined values of current error (**a**) and phase displacement (**b**) of distorted-current harmonics’ transformation by the developed iCT in its secondary winding with rated RMS value of current equal to 5 A for rated load equal to 5 W.

Verification tests for the transformation accuracy of the designed iCT 400A\5A\1A for distorted currents were performed with the same procedure and the measurement system presented in Figure 10. In the case of the iCT with two secondary windings (with a rated load of 5 W), both of them had to be separately examined. Moreover, since the 0.2S transformation accuracy class was expected, this iCT was also tested under conditions equivalent to 1% of the rated primary current. In Figure 20, the determined values of the current error and phase displacement of the distorted-current harmonics’ transformation by the developed iCT in its secondary winding with the rated RMS value of a current equal to 5 A for a rated load equal to 5 W are presented.

**Figure 21:**
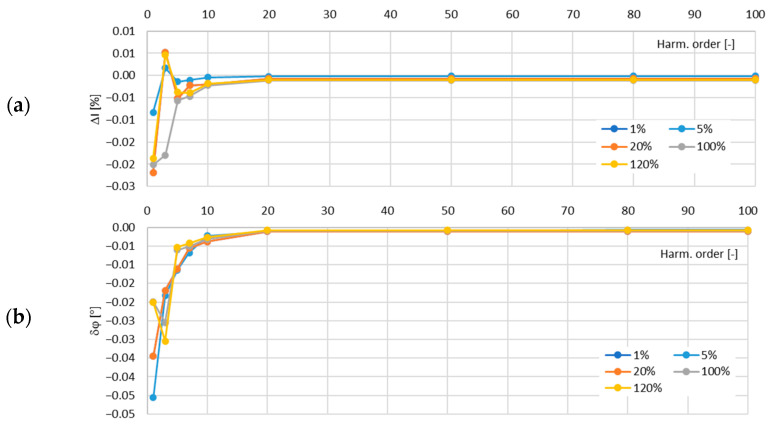
Determined values of current error (**a**) and phase displacement (**b**) of distorted-current harmonics’ transformation by the developed iCT in its secondary winding with rated RMS value of current equal to 5 A for rated load equal to 1 W.

Figure 21 shows the results of the wideband accuracy tests for the 1 W load of the 5 A secondary winding.

**Figure 22:**
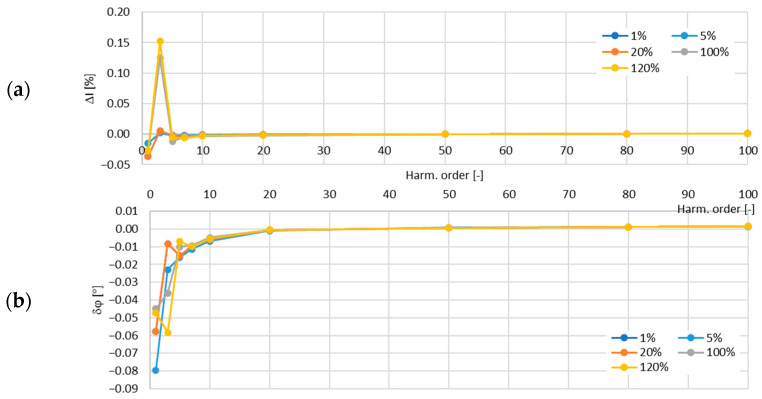
Determined values of current error (**a**) and phase displacement (**b**) of distorted-current harmonics’ transformation by the developed iCT in its secondary winding with rated RMS value of current equal to 1 A for rated load equal to 5 W.

In Figure 22, the determined values of the current error and phase displacement of the distorted-current harmonics’ transformation by the developed iCT in its secondary winding with the rated RMS value of a current equal to 1 A for a rated load equal to 5 W are presented. 

**Figure 23:**
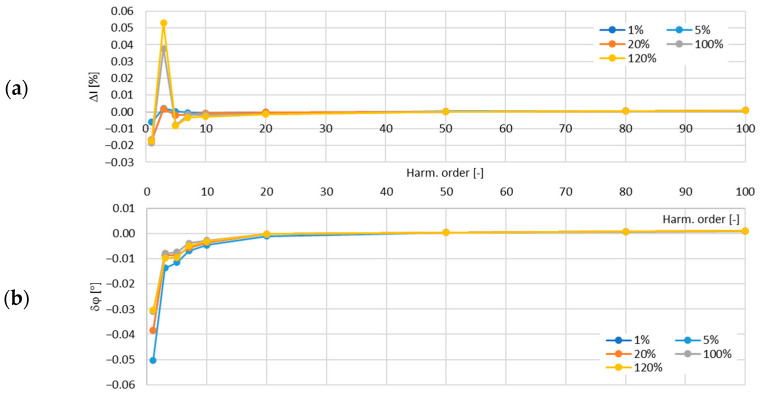
Determined values of current error (**a**) and phase displacement (**b**) of distorted-current harmonics’ transformation by the developed iCT in its secondary winding with rated RMS value of current equal to 1 A for rated load equal to 1 W.

Figure 23 shows the results for the 1 W load of the 1 A secondary winding.

Based on the presented results, it can be concluded that the developed iCT met the requirements of the accuracy class 0.2S of IEC 61869-2 in the frequency range of a transformed distorted current from 50 Hz up to 5 kHz. This results from the fact that the current error and phase displacement in the secondary winding with the rated current of 5 A reached the maximum values of ±0.15 and ±0.11° for the tested load range from 1 W to 5 W. The maximum permissible limiting values of ±0.2% for the current error and ±0.17° for the phase displacement were not exceeded. Moreover, the current error and phase displacement in the secondary winding with the rated current of 1 A reached the maximum values of ±0.15 and ±0.08° for the load range from 1 W to 5 W.

In accordance with the design and to ensure the same transformation accuracy, both windings should operate in the same conditions, taking into consideration the active power associated with the resistance of the secondary winding (the iCT’s equivalent circuit is presented in Figure 1). The length of the secondary winding with a rated current 5 A is approximately 8.3 m and the length with a rated current of 1 A is 43 m, taking into consideration the data provided in Table 2 and Figure 19 as well as the technical limitations of its manual winding. If the resistance of the wire with a diameter of 0.45 mm is 0.1075 Ω/m and the resistance of the wire with a diameter of 1.0 mm is 0.0218 Ω/m, then the resistance of the secondary windings with a rated current of 1 A is about 4.6 Ω and with a rated current of 5 A is 0.18 Ω. The active power dissipated in the winding is 4.6 W and 4.5 W, respectively. Therefore, the same operation conditions are ensured in the case of both designed iCTs’ secondary windings. This was confirmed by the results of the measurements presented in Figure 20, Figure 21, Figure 22 and Figure 23. The value of the phase error for the winding with a rated secondary current of 5 A under a rated load of 5 VA was maximally −0.08° for the fundamental component and −0.02° for the fifth-order harmonic, while for the winding with a rated secondary current of 1 A under a rated load of 5 VA, it was also maximally −0.08° for the main harmonic and −0.02° for the fifth-order harmonic. At the same time, the value of the current error for the winding with a rated secondary current of 5 A under a rated load of 5 VA was maximally −0.05% for the fundamental component and −0.01% for the fifth-order harmonic, while for the winding with a rated secondary current of 1 A under same load, it reached a maximum of −0.04% for the main harmonic and −0.01% for the fifth-order harmonic. Therefore, the diameter of the winding wire may be reduced, as long as the same current density is maintained it does not affect the values of the current error and phase displacement.

## 5. Conclusions

This research revealed the difficult and complex process of the development of a window-type inductive current transformer for the rated primary current with an RMS value of 400 A with two secondary windings with the rated RMS values of currents of 5 A and 1 A, designed for maintaining class 0.2S in a frequency range from 50 Hz to 5 kHz. In order to ensure the required wideband transformation accuracy, it is of importance to reduce the self-generation of the low-order higher harmonic into the secondary current. This may be achieved by a decrease in the load of the secondary winding in relation to the typically used cross-section of the magnetic core. Moreover, the diameter of the secondary winding wire should be oversized in order to decrease its resistance. These contribute to ensure the lowest possible operating point of the inductive current transformer in the non-linear magnetization characteristic of its magnetic core.

The results of our studies allowed us to select the most appropriate nanocrystalline magnetic core with an initial magnetic permeability of 100,000 for the construction of the designed wideband inductive current transformer. Its advantages in terms of transformation accuracy are greatest for the fundamental component of distorted current. However, for higher harmonics, it shows similar properties to magnetic cores with a higher initial magnetic permeability. It was demonstrated that, as far as more favorable magnetic properties also involves increased active power losses in the magnetic core, it is not an appropriate choice for the construction of inductive current transformers, especially for the transformation of a current with a main frequency equal to 50 Hz or 60 Hz.

The performed transformation accuracy tests of the developed inductive current transformer confirmed that the requirements of the 0.2S accuracy class defined in the standard IEC 61869-2 with the limiting values of the current error and phase displacement are met for the transformation of distorted-current harmonics’ in the entire frequency range from 50 Hz up to 5 kHz. In accordance with the design, these requirements for transformation accuracy are ensured by both secondary windings with rated RMS currents 5 A and 1 A. This was confirmed not only by the performed verification accuracy tests but also by the calculation of their operation conditions, taking into consideration the active power associated with their resistance resulting from the used wire’s diameter and its length.

## Figures and Tables

**Table 1:** The limiting values of the current error and phase displacement in accordance with the standard IEC 61869-2 for each defined accuracy class (ac. cl.) of the iCT.

ac. cl.	Current Error [%]					Phase Displacement [º]				
	at a Given Percentage of the Rated Primary Current									
	1%	5%	20%	100%	120%	1%	5%	20%	100%	120%
0.1	-	±0.4	±0.2	±0.1	±0.1	-	±0.25	±0.13	±0.08	±0.08
0.2	-	±0.75	±0.35	±0.2	±0.2	-	±0.5	±0.25	±0.17	±0.17
0.5	-	±1.5	±0.75	±0.5	±0.5	-	±1.5	±0.75	±0.5	±0.5
**0.2S**	**±0.75**	**±0.35**	**±0** **.2**	**±0.2**	**±0.2**	**±0.5**	**±0.25**	**±0.17**	**±0.17**	**±0.17**
0.5s	±1.5	±0.75	±0.5	±0.5	±0.5	±1.5	±0.75	±0.5	±0.5	±0.5

The class is highlighted as the iCT is designed to meet 0.2S accuracy class.

**Table 2:** Characterization of four tested window-type iCT models with Nano toroidal magnetic cores.

No.	Dimensions			Initial Permeability [-]	Number of Turns [-]
	Outer Diameter [mm]	Inner Diameter [mm]	Height [mm]		
1	61	35	20	200,000	50
2				150,000	
3				100,000	
4				40,000	

## Data Availability

Data are provided within this manuscript.
